# Latanoprost-Loaded Nanotransfersomes Designed for Scalp Administration Enhance Keratinocytes Proliferation

**DOI:** 10.1021/acs.molpharmaceut.2c00796

**Published:** 2022-12-12

**Authors:** Eloy Pena-Rodríguez, Laura García-Vega, Maria Lajarin Reinares, Marçal Pastor-Anglada, Sandra Pérez-Torras, Francisco Fernandez-Campos

**Affiliations:** † Laboratory Reig Jofre, R&D Department, 08970Sant Joan Despi, Barcelona, Spain; ‡ Molecular Pharmacology and Experimental Therapeutics, Department of Biochemistry and Molecular. Biomedicine, Institute of Biomedicine (IBUB), University of Barcelona (IBUB), 08028Barcelona, Spain; § Biomedical Research Networking Center in Hepatic and Digestive Diseases (CIBEREHD), Carlos III Health Institute, 28029Madrid, Spain; ∥ Sant Joan de Déu Research Institute (IR SJD-CERCA) Esplugues de Llobregat, 08950Barcelona, Spain

**Keywords:** nanotransfersomes, human skin, pig skin, latanoprost, hair follicle, HaCaT, sonication, confocal laser microscopy, biodistribution

## Abstract

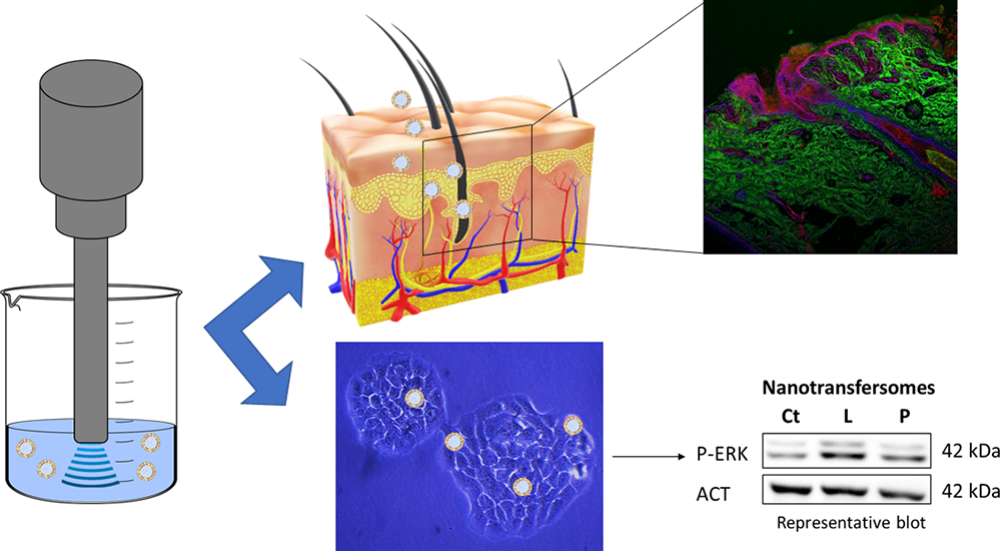

Latanoprost (LAT) has been shown to have a hypertrichotic effect, which makes it a promising candidate for alopecia treatments. For the first time, LAT has been encapsulated in nanotransfersomes in order to increase its efficacy. *Ex vivo* skin biodistribution was studied by confocal laser microscopy both in human scalp and pig skin. Results showed that nanotransfersomes increase the penetration of two different fluorochromes, with similar patterns in both species, compared with fluorochrome solutions containing no nanotransfersomes. Nanotransfersomes were stable under accelerated conditions (40 °C/75% RH) and long-term conditions (25 °C/60% RH) for up to 1 year, with no differences in vesicle size and polydispersity when LAT was loaded. Nanotransfersomes increased the LAT cell proliferation effect in HaCaT cell via MAPK signaling pathway. Collectively, our results demonstrate LAT-nanotransfersomes formulation could be a promising therapy for hair growth disorders.

## 1 Introduction

Nanoliposomes are self-assembling vesicular systems with a diameter below 100 nm. They encapsulate hydrophilic molecules in their aqueous core as well as hydrophobic or amphiphilic molecules, which are intercalated in their lipid membrane. Their small size allows them to permeate across different biological membranes and barriers, increasing the bioavailability of different molecules. Transfersomes are a subtype of liposomes that contain edge activators (usually surfactants) in their membranes, which increase their deformability compared with traditional liposomes. This deformability increases dermal penetration as they can extrude across skin pores. In addition, the edge activators can act as permeation enhancers, since they can modify the solubility and packaging of skin lipids.^1^ Transfersomes with a diameter below 100 nm are also called nanotransfersomes.^2^

There are different techniques for the production of liposomes. The most classical method is the Bangham method,^3^ also known as the thin-film hydration method, consists of dissolving lipids in an organic phase, removing the organic solvent by evaporation to form a lipid layer, and rehydrating the layer with an aqueous medium under agitation. Sometimes an additional step is required, such as sonication or extrusion, to reduce size and/or vesicle lamellarity.^4^ The use of classic organic solvents, such as chloroform or dichloromethane, limits its scalability and sustainability and could give rise to toxicological concerns. On the other hand, ultrasonication methods produce liposomes of different sizes and with higher polydispersity indexes (PdI), which limit their use in injectable systems that require monodispersion. However, they have acceptable values for topical application. The sonication energy supplied by the system produces cavitation.^5^ When liquids are sonicated at high intensities, the acoustic waves propagating through the liquid medium produce alternating cycles of high and low pressures at a frequency-dependent rate. This method creates small vacuum bubbles within the liquid, and when they can no longer absorb energy, they collapse violently causing a reduction in the size of liposomes. This phenomenon is called cavitation. This technique is one of the most promising techniques for the industrial scale-up of liposomes.

Latanoprost (LAT) is a prostaglandin F2 alpha (PGF2α) analogue commonly used to treat glaucoma and ocular hypertension.^6^ Most of its adverse effects are classified as mild intensity, such as eyelash hypertrichosis.^7^ Encouraging results have been observed in animal models and clinical trials for alopecia, suggesting prostaglandin signaling could play a role in hair proliferation.^8,9^ However, little is known about its cellular mechanism to increase hair growth.

Several studies have encapsulated LAT into liposomes to extend drug release and improve the therapeutic index in glaucoma and related diseases.^300–303^ There is no information about LAT encapsulation in nanotransfersomes for topical administration as a potential tool to treat hair disorders.

In previous studies, Pena-Rodríguez et al. succeeded in obtaining epidermal targeting by loading retinyl palmitate in transfersomes with a diameter between 300 and 400 nm.^10^ In this case, smaller vesicles (nanotransfersomes) were selected to increase the skin absorption of the drug and to deliver it to the deep terminal hair follicles of the human scalp in the dermis.

The aim of this work was to produce LAT-loaded nanotransfersomes for skin application. *In vitro* LAT drug release and *ex vivo* nanotransfersome biodistribution in human scalp explants were studied. Finally, the ability of LAT to induce keratinocyte proliferation was analyzed *in vitro*, and the possible cellular pathways involved were also studied.

## 2 Materials and Methods

### 2.1 Materials

α-Tocopherol (Merck KGaA, Darmstadt, Germany), phosphatidylcholine (Lipoid, Ludwigshafen, Germany), cholesterol (Merck KGaA, Darmstadt, Germany), LAT (Fagron, Barcelona, Spain), Tween 80 (Croda Iberica S.A., Barcelona, Spain), ethanol absolute (Scharlab S.L., Barcelona, Spain), and purified water (in-house) were used to formulate the nanotransfersomes. Methanol (Scharlab S.L., Barcelona, Spain), 1,2-dioleoyl-3-[16-*N*-(lissamine rhodamine B sulfonyl) amino]palmitoyl-*sn*-glycerol (LRB) (Avanti Polar Lipids, Alabaster, AL), Hoechst 33342 (Merck KGaA, Darmstadt, Germany), phosphate-buffered saline (PBS) (Merck KGaA, Darmstadt, Germany), paraformaldehyde (Merck KGaA, Darmstadt, Germany), sodium fluorescein (Merck KGaA, Darmstadt, Germany), uranyl acetate (Electron Microscopy Sciences, Hatfield, England), and optimal cutting temperature (OCT) compound (Thermo Fisher Scientific, Waltham, MA) were used to perform the different analyses.

For cell culture, the human keratinocyte cell line HaCaT (Cell Line Service, Eppelheim, Germany)^11^ was maintained in Dulbecco’s modified Eagle’s medium (DMEM)-high glucose (4.5 g/L) with l-glutamine (Life Technologies, Paisley, UK) supplemented with 10% (v/v) fetal bovine serum (FBS) (Life Technologies, Paisley, UK) and 50 U/mL/20 μg/mL penicillin/streptomycin (Life Technologies, Paisley, UK). Proliferation assays were performed by CCK8 assay (Merck KGaA, Darmstadt, Germany).

Western blot analysis was carried out with PVDF Immobilon-P transfer membrane (Merck KGaA, Darmstadt, Germany), Bio-Rad mini protean II (Bio-Rad, Hercules, CA), Bio-Rad Clarity Western ECL substrate (Bio-Rad, Hercules, CA), and iBright Imaging Systems (Invitrogen, Waltham, MA). The antibodies used were: rabbit anti-P(S473)-AKT (9271, Cell Signaling Technology, Danvers, MA), goat anti-AKT (sc1618, Santa Cruz Biotechnology, Dallas, TX), rabbit anti-p-ERK (9101, Cell Signaling Technology, Danvers, MA), rabbit anti-ERK (V114A, Promega, Madison, WI), rabbit anti-Actine (A2066, Merck KGaA, Darmstadt, Germany), rabbit anti-goat (P0449, Dako, Glostrup, Denmark), goat anti-rabbit (172–1019, Bio-Rad, Hercules, CA), or goat anti-mouse (172–1011, Bio-Rad, Hercules, CA).

### 2.2 Formulations

Nanotransfersomes were manufactured by the sonication method.^12,13^ For the Placebo-nanotransfersomes, α-tocopherol (0.01% w/w), cholesterol (0.01% w/w), phosphatidylcholine (0.73% w/w), hydrogenated phosphatidylcholine (0.20% w/w), and Tween 80 (0.05% w/w) were dissolved in ethanol (10% w/w). Then, milli-Q water (qs 100% w/w) was added to the organic phase at 500 rpm (paddle stirring) until complete homogenization. LAT-nanotransfersomes were produced in the same way, by adding LAT (0.005% w/w) to the ethanolic solution.

Fluorescent nanotransfersomes were produced to study skin biodistribution. Fluorescein (0.1% w/w) was selected as the hydrophilic fluorochrome, which was encapsulated in the aqueous core of the vesicle. It was added to the water phase. LRB (0.0003% w/w) was added to the ethanolic phase, with this hydrophobic fluorochrome inserted into the lipid bilayer of the nanotransfersome. After its production, the formulation was dialyzed overnight (Slide-A-Lyzer 10-kDa cutoff cassettes (Thermo Fisher Scientific, Barcelona, Spain)) to remove and quantify (section 2.5) the unloaded fluorescein. Free fluorochrome solution was produced at equivalent concentrations of LRB and the encapsulated fluorescein with the addition of 0.05% Tween 80 to solubilize LRB.

An UP400st ultrasonicator (Hielscher Ultrasonics, Germany) was used with an amplitude of 40% for 5 min to produce the nanotransfersomes.

### 2.3 Physicochemical Characterization

Hydrodynamic size (*Z*-average) and the PdI were studied with dynamic light scattering (using a Malvern Zetasizer Nano ZS) (Malvern Panalytical, Malvern, UK). Dilutions of 1:10 in water were used for the measurements.

Nanotransfersome particle size of the selected formulation after scale-up was studied with transmission electron microscopy (TEM) using a Jeol JEM 1010 100kV electron microscope (Jeol, Tokyo, Japan). TEM grids were coated with Formvar and incubated with a 1:10 nanotransfersome dilution in milli-Q water for 1 min at room temperature. The grids were then washed with water and stained with a 2% w/w uranyl acetate solution for 1 min at room temperature. Afterward, nanotransfersomes were dried overnight and analyzed.

### 2.4 Nanotransfersomes Stability Studies

Nanotransfersomes were subjected to stability studies in chambers at 25 ± 2 °C/60 ± 5% relative humidity (RH) (long-term conditions) or 40 ± 2 °C/75 ± 5% RH (accelerated conditions) and packaged in hermetically sealed glass vials. The *Z*-average and PdI were characterized by DLS, according to section 2.3, at time points 0, 0.5, 1, 3, and 6 months for accelerated conditions (40 °C/75% RH) and at 0, 0.5, 1, 3, 6, 9, and 12 months for long-term conditions (25 °C/60% RH).

### 2.5 Drug Analysis and Encapsulation Efficiency

LAT was analyzed by high-performance liquid chromatography (HPLC). The assay was performed in a Waters 2695 HPLC equipped with a Waters 2996 detector (Waters Corporation, Milford, MA). Briefly, a mobile phase (acetonitrile:water, 60:40 v/v) flowed isocratically through a Xterra MS (reference 186000494) C18 HPLC column (250 × 4.6 mm, 5 μm) (Waters Corporation, Milford, MA) at a flow rate of 1 mL/min. The injection volume was 20 μL, and the wavelength was 210 nm. The sample and column were maintained at 25 °C. The limit of quantification (LOQ) of the analytical technique was set at 0.1 ng/mL.

Fluorescein was quantified with a UV spectrophotometer (VICTOR Multilabel Plate Reader, PerkinElmer, Waltham, MA) at the wavelength of 485 nm.

Encapsulation efficiency, according to the indirect method, was calculated according to eq 1.
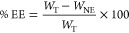
1where *W*_NE_ is the amount of drug quantified in the filtrate (drug not encapsulated) and *W*_T_ the drug quantified in the total formula. Liposomes were centrifuged in 100 kDa Amicon Ultra units (Merck KGaA, Darmstadt, Germany) at 4500 rpm for 30 min.

### 2.6 Latanoprost Drug Release

*In vitro* LAT release (*n* = 6) from nanotransfersomes was studied in vertical Franz diffusion cells (Vidrafoc, Barcelona, Spain) with an effective diffusional area of 1.54 cm^2^. The receptor compartment was filled with a pH 7.4 PBS solution, with 5% hydroxypropyl-beta-cyclodextrin (to maintain sink conditions) and kept at 32 °C and continuous stirring at 500 rpm. 1 mL of LAT-nanotransfersomes was loaded in the donor compartment, which was separated from the receptor compartment by a dialysis membrane (Spectrum Chemical, New Brunswick, NJ) with a pore size cutoff value of 12–14 kDa. Samples of 300 μL were taken regularly for up to 24 h and analyzed by the HPLC method, as described in section 2.3.

### 2.7 *In Vitro* Penetration Tests with Full-Thickness Pig and Human Scalp

Human scalp was purchased from Biopredic (Saint Grégoire, France), which has authorization for the collection, processing and sectioning of human biological samples for research purposes. Samples were remnants from surgeries complying with the French law CSP1245–2, with informed consent provided by the patient, who remained anonymous and did not receive financial reward or publicity. The donor was a 58-year-old Caucasian female.

Pig skin was obtained at the time of sacrifice from a local abattoir (Barcelona, Spain). The skin was cleaned with sterile saline solution and transported to the laboratory at 4 °C in saline solution. Both human and pig full-thickness skin pieces were defatted (with a scalpel) and frozen at −20 °C until use.

The permeation experiment was performed in Franz diffusion cells with an effective diffusion area of 0.196 cm^2^. Skin pieces were placed between the donor compartment and receptor compartment, which was filled with PBS, pH 7.4, and 4% albumin at 32 °C and stirred at 500 rpm. Transepidermal water loss (TEWL) was recorded with a vaporimeter device (Delfin Technologies, Kuopio, Finland) to check skin integrity before the experiment. An amount of 76 mg of each formulation were placed into the donor compartment (fluorescent nanotransfersomes and free fluorescent solutions).

After 18 h of permeation, the skin sections treated with the fluorescent formulations were washed with PBS, cut with a scalpel into pieces of about 0.5 cm^2^, and fixed in 4% w/w paraformaldehyde solution for 5 min. Then, the skin samples were incubated in aqueous solutions of increasing sucrose concentration (5, 15, and 25% w/w) for 15 min in each solution. They were then placed in plastic molds and dipped in OCT to cut on a Leica CM 3050 S cryostat (Leica Biosystems, Barcelona, Spain) into 50 μm thicknesses. The slices were collected on polylysine-coated slides and washed with PBS and 0.05% Tween 20 (TPBS) for 5 min to remove the OCT and permeabilise the samples. On the day of observation, sections were incubated with 15 μL of Hoechst solution (2 μg/mL) for 10 min and washed with TPBS to stain the cell nuclei.

### 2.8 Confocal Laser Microscopy

The samples were analyzed under a confocal microscope (Leica Microsystems, Wetzlar, Germany). The emission laser wavelengths were 570, 500, and 525 nm, and the excitation wavelengths were 561, 488, and 405 nm for LRB, fluorescein and Hoechst, respectively. About 20 Z-planes were obtained per image, separated by a 3 μm step. Composites of the different planes were created, in terms of the brightest point for each pixel, through the ImageJ tool Z-stack (ImageJ2 v2.35, National Institutes of Health, Bethesda, MD). 3D projections of each set of planes were obtained using the 3D project tool and the brightest point projection method with ImageJ software (available in the Supporting Information). A skin blank was processed in the same way as the test samples to quantify skin autofluorescence. Mean intensity of the blank was measured with polygon selection and measure tools in the ImageJ software and was subtracted from the intensity of the red and green fluorescence of the samples. Linear segments were drawn to analyze the intensity profiles as a function of the depth (in μm) with the multichannel plot profile tool in ImageJ.

### 2.9 Cell Culture and Treatments

HaCaT cells were cultured in a 37 °C humidified incubator in an atmosphere of 5% CO_2_. PCR amplification was carried out every 14 days to confirm the absence of mycoplasma contamination.

In all assays, cells were starved for 24 h by replacing their supplemented DMEM with serum free medium before any treatment. Then, they were treated with LAT-free, LAT-nanotransfersomes or Placebo-nanotransfersomes.

### 2.10 Cytotoxicity: CCK8 Assay

Cells were seeded at appropriate densities into 96-well plates for CCK8 assay (7 × 10^3^ cells/well). They were starved for 24 h and treated for 72 h previously performing a CCK8 assay following the manufacturer’s instructions.

Cytotoxicity assays were performed in quintuplicate and repeated at least three different times to perform statistical calculations. All values indicate the mean ± standard error of the mean (SEM); the number of independent experiments is denoted by *n*. Data were compiled and analyzed in GraphPad Prism (RRID:SCR_002798). Statistical analysis was carried out using the one-way ANOVA test and Dunnett’s multiple comparisons test. Adjusted statistical significance denoted: no significant (ns), *, *p* < 0.05; **, *p* < 0.01; ***, *p* < 0.005; and ****, *p* < 0.001.

### 2.11 Western Blot

For protein extraction, 0.5 × 10^6^ cells/well were seeded into 6-well plates. They were harvested in 100 μL ice-cold phosphorylated lysis buffer (20 mM Tris, 150 mM NaCl, 10 mM EDTA, 10 mM sodium pyrophosphate, 2 mM orthovanadate, 100 mM NaF, 1 mM β-glycerophosphate, and 1% NP40) per well. Total protein (20–50 μg depending on the protein to be studied) was separated on 10% (w/v) sodium dodecyl sulfate-polyacrylamide gels and transferred to a PVDF membrane for 70 min at 180 mA. Membranes were blocked with 5% (w/v) skimmed milk in Tris-buffered saline-Tween (TBS-T) (200 mM Tris, 1.5 mM NaCl, and 0.1% (v/v) Tween 20) and probed with primary antibodies at 4 °C overnight. Compatible horseradish peroxidase (HRP)-conjugated secondary antibodies were used at dilution 1:2000, for 1 h, at room temperature. Membranes were incubated for 1 min with Bio-Rad Clarity Western ECL substrate (1:1 Luminol/Enhancer solution/peroxide solution) in the dark and visualized with iBright Imaging Systems. Band density semiquantification was performed with Invitrogen iBright Analysis Software, Desktop Version 5.0.1.

## 3 Results and Discussion

### 3.1 Formulations and Stability Studies

The results of the DLS characterization of placebo-nanotransfersomes, LAT-nanotransfersomes, and fluorescent-nanotransfersomes (LRB-fluorescein-loaded) after production of the laboratory-scale formulations are shown in Table 1. The inclusion of LAT into the nanotransfersomes did not affect either size or the PdI. Nanotransfersome size and PdI remained below 100 nm and was 0.3, respectively, in all cases.

**Table 1:** Nanotransfersome Size and PdI Values of the Different Formulations

formulation	size (nm)	PdI	%EE
placebo-nanotransfersomes	60.48	0.206	NP
LAT-nanotransfersomes	67.22	0.181	100%
fluorescent-nanotransfersomes	71.92	0.190	24.11%

The encapsulation efficiency for LAT was 100% since the *W*_NE_ values were below the LOQ after ultrafiltration. On the other hand, the %EE of fluorescein was low (24.11%), as would be expected for hydrophilic compounds. The free fluorescein solutions for the confocal experiments were produced at the same concentration (0.024% w/w). Placebo-nanotransfersomes were subjected to long-term conditions (25 °C/60% RH) for 1 year and accelerated conditions (40 °C/75% RH) for 6 months in the stability studies.

**Figure 1:**
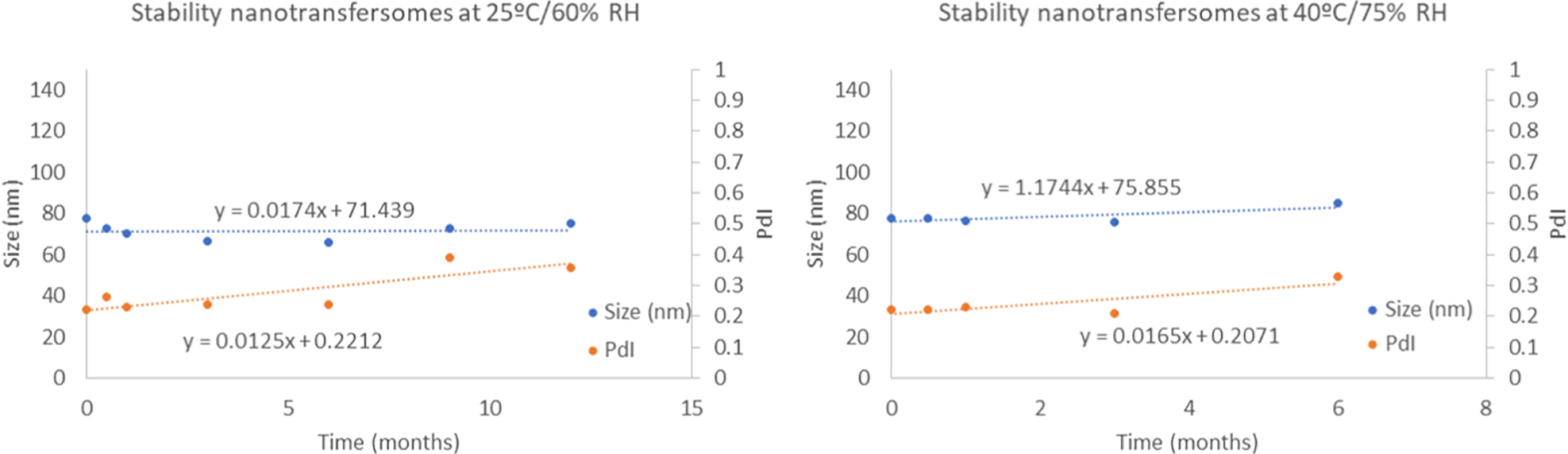
Size and the PdI of placebo nanotransfersomes under long-term (25 °C/60% RH) and accelerated conditions (40 °C/75% RH).

The *Z*-average and PdI values are shown in Figure 1. Regression slopes and the statistical significance were also obtained.

### 3.2 Latanoprost Drug Release

**Figure 2:**
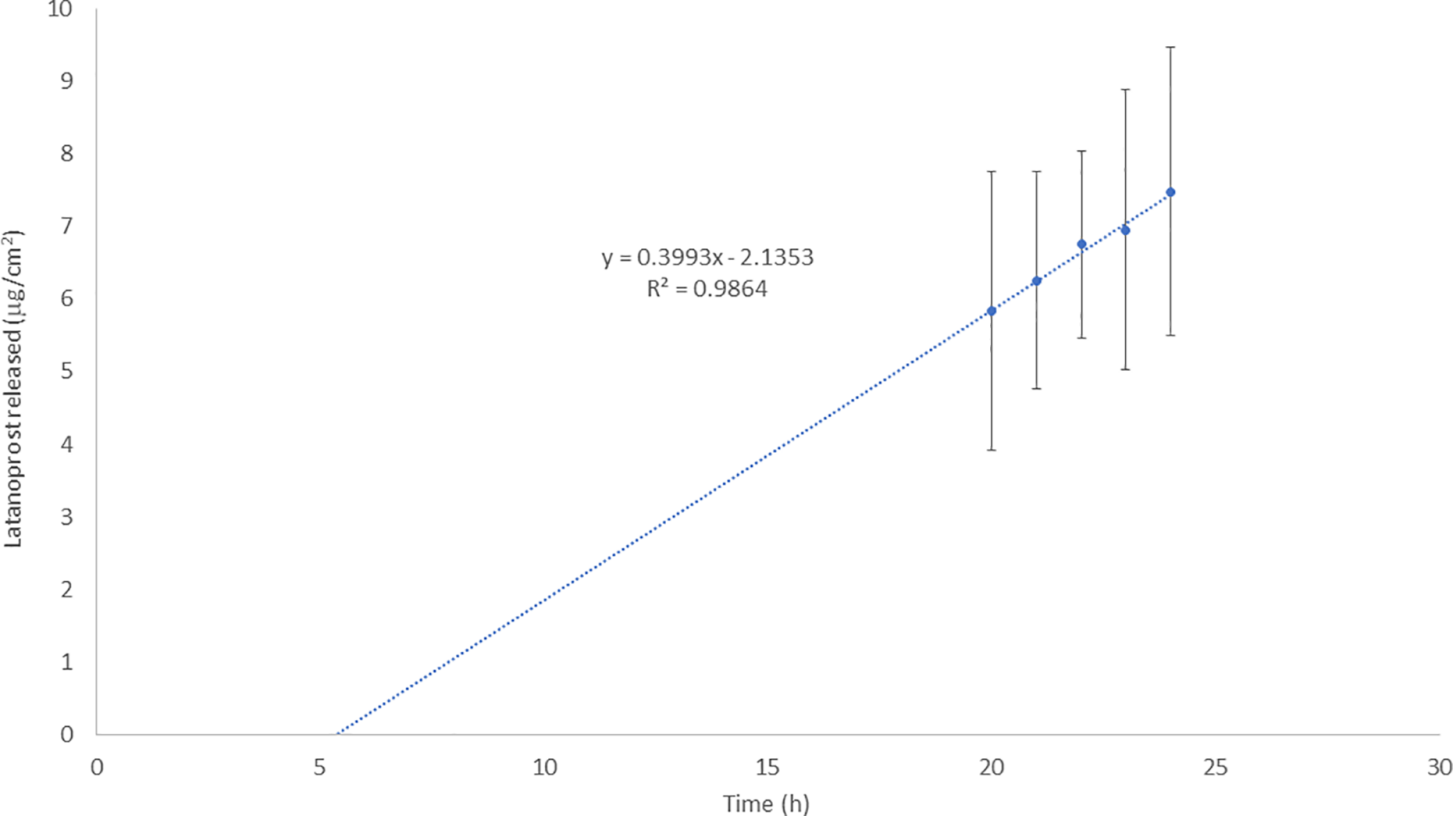
Release profile of LAT from nanotransfersomes and the regression equation. Points represent the empirical mean and standard deviation. Line is the estimated regression line.

Figure 2 shows the release profile of LAT-nanotransfersomes after 24 h of analysis. During the first hours, no LAT was observed in the receptor medium (<LOQ). Only after 20 h, small amounts of LAT were found, with a very low increase over time. The slow release could be related to the high lipophilicity of LAT, which probably is a structural part of the nanotransfersome membrane. Vesicles could act as a drug reservoir, which could be useful in reducing the administration frequency. Drug release over time showed a linear pattern, compatible with a controlled release profile, with a release constant equivalent to 0.3993 μg/cm^2^/h. The total LAT released at 24 h corresponded to 23.46% of the loading dose in the donor compartment.

### 3.3 Confocal Fluorescence Microscopy to Study the Skin Biodistribution of Nanotransfersomes

The skin penetration-promoting effect of nanotransfersomes has been described in the literature. Many of these studies were performed on pig skin, which is one of the more similar models compared to human skin. Even so, there are several differences between pig and human skin, such as the different thicknesses of the stratum corneum and epidermis and the lack of sebaceous and sweat glands in pig skin. This could be important in follicular targeting studies, especially for products applied to the scalp. To evaluate the possible differences in permeability between the two species and to demonstrate the higher permeability properties of the studied nanotransfersomes, penetration studies of fluorescent vesicles were performed.

Two different fluorophores were coencapsulated, sodium fluorescein (NaFl) and LRB. NaFl (green fluorophore) simulates the behavior of a hydrophilic drug and was encapsulated in the aqueous core of the nanotransfersomes. LRB (red fluorochrome) simulates the behavior of a lipophilic drug due to the presence of C16 hydrocarbon tail which is encapsulate on the lipid membrane of the vesicles. Free solutions of both fluorophores were used as controls. Hoechst staining was performed on the histological sections to observe the cell nuclei (in blue) and improve visualization.

**Figure 3:**
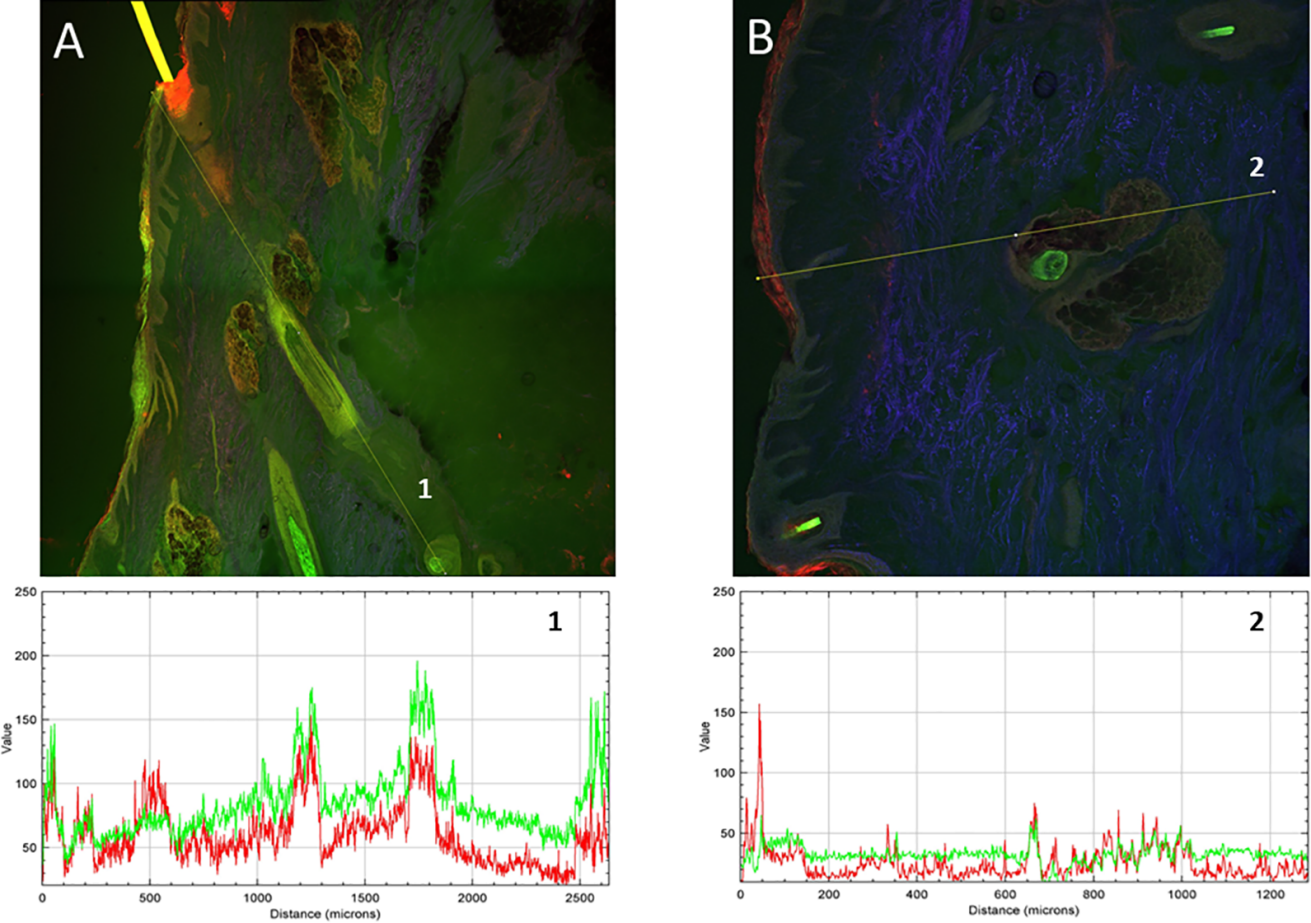
Confocal fluorescence microscopy images of human scalp cross sections. Green color corresponds to NaFl and red to LRB. A) NaFl-loaded LRB-labeled nanotransfersomes. B) Free NaFl and LRB control solution. Lines 1 and 2 correspond to multichannel intensity plot profiles as a function of the depth (μm). The images were captured using 10× magnifications. 3D projections of each image are available as Videos S1 and S2.

The biodistribution results are shown in Figures 3 (human scalp) and 4 (pig skin). The yellow color corresponds to the colocalization of NaFl and LRB. Representative images of each permeation are reported.

**Figure 4:**
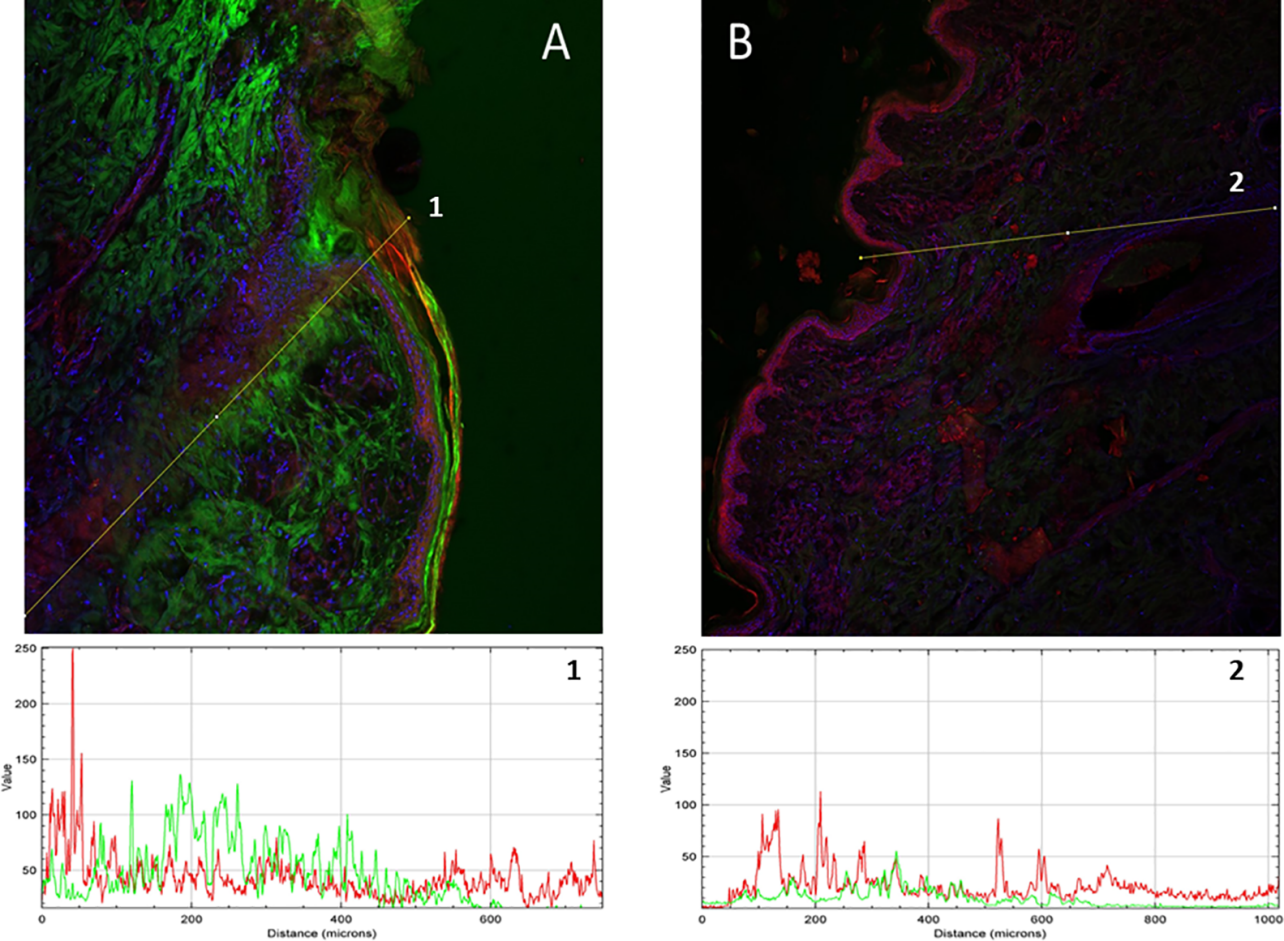
Confocal fluorescence microscopy images of pig skin cross sections. Green color corresponds to NaFl and red to LRB. A) NaFl-loaded LRB-labeled nanotransfersomes. B) Free NaFl and LRB control solution. Lines 1 and 2 correspond to multichannel intensity plot profiles as a function of the depth (μm). The images were captured using 10× magnifications. 3D projections of each image are available as Videos S3 and S4.

Comparing the images and the intensity profiles of the lines plotted in Figure 3, differences were observed in the biodistribution of the fluorophores. A clear absorption-promoting effect of the nanotransfersomes for NaFl was observed (Figure 3A, line 1), while nonvehiculated NaFl (Figure 3B, line 2) showed very low skin penetration, with its fluorescence intensity remaining below 50 AU even in the most superficial layers of the skin. The green intensity in Figure 3A line 1, even in the deepest layers of the skin, reached levels above 150 AU at depths of up to 2500 μm, i.e., an increase of 300% compared to the NaFl control solution. The absorption of LRB was also higher in the presence of nanotransfersomes. In the intensity profile in Figure 3A line 1, values above 100 AU can be observed at depths of 1250 and 1750 μm. Nonvehiculated LRB (Figure 3B, line 2) showed lower intensities that were below 50 AU.

The same effect was found in experiments on pig skin (Figure 4). Clear differences were also observed in the cutaneous biodistribution between the vehiculated fluorophores and the control solution. As for NaFl, similar to that observed in the human scalp, the control solution (Figure 4B) showed no signs of NaFl penetration into the stratum corneum or the epidermis, nor in the deeper layers. However, Figure 4A shows strong coloration corresponding to NaFl from the superficial layers to the dermis. When comparing the intensities, the intensity was below 25 AU practically throughout the whole tissue for the control solution (Figure 4B, line 2), while it was between 50 and 100 AU at depths of up to 400 μm for the NaFl vehiculated in the nanotransfersomes (Figure 4A, line 1).

The tiny size (<100 nm), the presence of surfactants and the high deformability of these vesicles explain why nanotransfersomes were able to increase the penetration of encapsulated compounds in their core to deep sections of the dermis. These vesicles are able to enhance the dermal penetration of both hydrophilic and lipophilic compounds out of the log *P* range (1–3),^10,14^ which is the optimal lipophilicity for skin absorption.^15^

Due to the different physicochemical properties (hydrophilicity of NaFl vs hydrophobicity of LRB), the arrangement of the fluorochromes inside the nanotransfersomes will possibly be different (aqueous core vs lipid membrane respectively), and consequently their cutaneous biodistribution will also be different. It is probable that, once in contact with the interkeratinocytic lipids, the nanotransfersomes destructure causing the release of their aqueous core. This would explain that the nanotransfersomes allowed the NaFl to pass through the stratum corneum membrane (lipid-rich area) and once released, the molecule was able to diffuse through the epidermis and dermis.

Hosny et al. analyzed the scalp *ex vivo* permeation of Finasteride loaded nanotransfersomes observing an increased permeation of nanotransfersomal formulation,^16^ but the biodistribution of the nanoparticles was not studied. Ahmed and Rizq studied the *ex vivo* biodistribution of a Finasteride nanotransferosomal gel through fluorescence microscopy, but the experiments were performed in rat abdominal skin.^17^ Several studies have been published showing that rat skin is more permeable than human skin.^18^

Comparing the permeation in pig and human skin, the intensity of both fluorochromes was similar in the control and test samples. Pig skin remained a good surrogate of human scalp skin despite the lack of sebaceous glands. This is an important feature to consider when lipophilic molecules are studied, as can be seen in Figure 3A, which shows an accumulation of LRB in the sebaceous glands.

Androgen-sensitive areas show significant enlargement of sebaceous glands, compared to androgen-insensitive scalp areas.^19^ These glands play an important role in the physiology of the skin (hydrolipidic content, skin pH regulation, follicular microbiome) and due to their lipid nature, they can act as a reservoir for lipophilic molecules. They are also attracting growing interest due to the stem cells they contain.^20^ Due to the mechanism of action of LAT, a greater efficacy could be expected in androgenic alopecia due to the pilosebaceous unit targeting, which should be tested in future clinical trials.

### 3.4 Latanoprost Proliferation Effect and Cell Signaling

**Figure 5:**
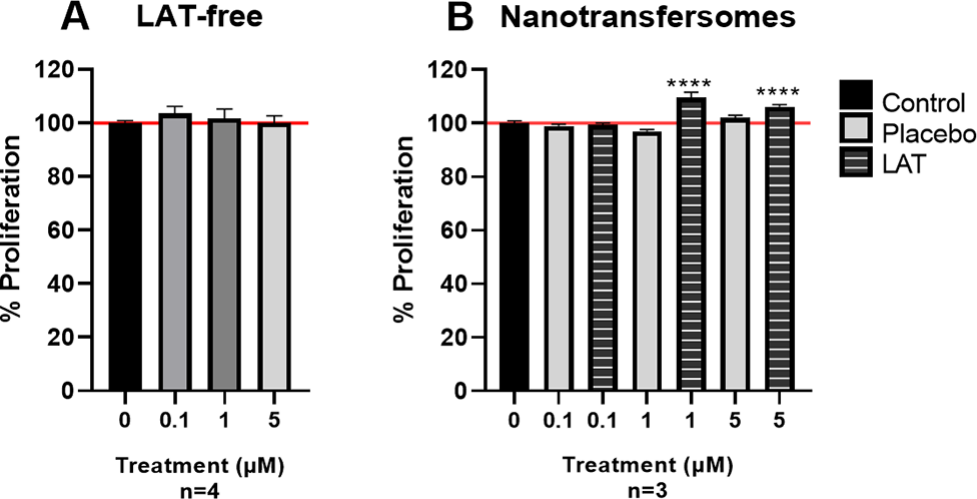
LAT effects on HaCaT cells proliferation. A) HaCaT cells exposed to LAT-free 1 μM for 72 h. B) HaCaT cells treated with LAT/Placebo-nanotransfersomes or preservatives for 72 h. Data are represented as the mean ± SEM of the cell proliferation percentage, referring to untreated control (horizontal line). Statistical significance was determined with one-way ANOVA test and Dunnett’s multiple comparisons test; ****, *p* < 0.001.

The cellular mechanism of LAT-induced hair growth remains unknown, given that few data have been published detailing LAT-treated cell culture. HaCaT cells were chosen to determine the LAT effects, whether free or encapsulated, on cell proliferation. They are widely used as skin keratinocytes *in vitro* model as they preserve differentiation capacity. LAT-free (at 1 μM) did not significantly affect HaCaT cell proliferation (ns, Figure 5A). Similarly, Stjernschantz’s group did not find a proliferative effect of LAT on keratinocytes.^21^ Considering that hair follicle is formed by different cell types such as dermal papilla cells and/or fibroblast, the study on cell proliferation could be extended to them, although the same authors did not find it in fibroblasts either.^21^ Next, HaCaT cells were treated with LAT- and Placebo-nanotransfersomes at final concentrations of 5, 1, and 0.1 μM. LAT-nanotransfersomes significantly increased proliferation compared to control (5 μM = 9.53%, ****, *p* < 0.001 and 1 μM = 6.18%, ***, *p* < 0.005), while Placebo-nanotransfersomes did not affect cell proliferation (ns, Figure 5B).^21^

LAT is very hydrophobic which hinders its administration in the aqueous medium. However, its encapsulation could enhance cell targeting as evidenced by Oliveira’s lab, who obtained similar results. Their coencapsulated Minoxidil and LAT in nanostructured lipid carriers significantly increased keratinocytes proliferation compared to untreated controls, whereas LAT-free and/or Minoxidil-free did not.^22^ Notably, in a clinical trial in which 123 patients were treated with Minoxidil, LAT, and Minoxidil + LAT combinations, they found greater efficacy with Minoxidil and LAT treatments administered separately rather than combined.^23^

The canonical mitogen-activated protein kinase (MAPK) and phosphoinositide 3 kinase (PI3K) pathways are activated by multiple mitogens and regulate cell proliferation in various cell types with keratinocytes being no exception.^24,304^*In vitro* assays have demonstrated that proliferative HaCaT cells present activated MAPK and/or PI3K pathways depending on the treatment.^25^ These two pathways are obviously involved in hair growth as well.^26,27^ Additionally, LAT regulates proliferation and survival via MAPK and PI3K pathways in several cell models. It promoted outgrowth of retinal ganglion cells-5 by activating p-AKT while rescuing retinal neuro-glial cells from apoptosis via p-ERK.^28,29^

**Figure 6:**
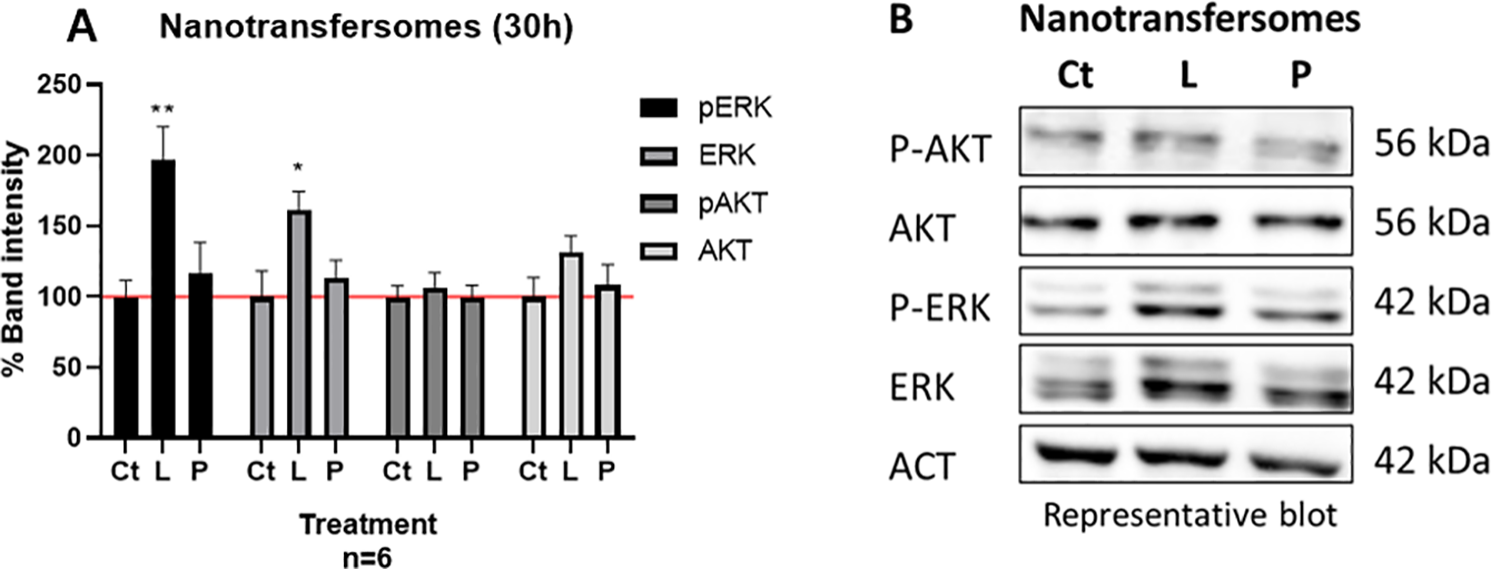
LAT-nanotransfersomes promote cell proliferation signaling. A) Semiquantification of band density. Data are represented as the mean ± SEM of the cell proliferation percentage, referring to untreated control (Ct, horizontal line). Statistical significance was determined with One-way ANOVA test and Dunnett’s multiple comparisons test; *, *p* < 0.05, and **, *p* < 0.01. B) Representative blot of nanotransfersomes effect on HaCaT cells. Ct: control, L: LAT-nanotransfersomes, P: Placebo-nanotransfersomes. Actin (ACT) was used as a loading control.

Consequently, cell signaling was studied by Western blot. Initially, proliferation signaling was studied after 6 and 24 h, but no significant change was observed (data not shown). However, by lengthening the incubation time to 30 h, cell proliferation induction was detected (Figure 6). A significant increase in p-ERK and ERK was observed after the 30 h LAT-nanotransfersomes treatment (p-ERK = 96.94% **, *p* < 0.01 and ERK = 61.13% *, *p* < 0.05, Figure 6A). These results are in line with the slow-release kinetics observed in LAT-nanotransfersomes (Figure 2), in which after 24 h only 23.46% of the active ingredient was released. Placebo-nanotransfersomes did not promote changes in cell signaling (ns, Figure 6A).

Overall, LAT-nanotransfersomes are able to induce keratinocyte proliferation overcoming latanoprost hydrophobicity. Moreover, the different cell types that form the hair follicle intercommunicate to modulate cell proliferation. In this sense, the use of more complex models such as cocultures of keratinocytes, dermal papilla cells or fibroblasts could be of interest to better resemble the hair follicle.^30^

## 4 Conclusion

Nanotransfersomes were successfully developed as encapsulation systems of both hydrophilic and lipophilic small molecules (LAT, NaFl, and LRB). The vesicles had a particle size below 100 nm and a PdI value below 0.3. The formulation was stable under long-term conditions (25 °C/60% RH) for 1 year and underaccelerated conditions (40 °C/75% RH) for up to 6 months. The vesicle showed a slow-release pattern. Furthermore, the nanotransfersomes promoted the skin penetration of the loaded fluorochromes in both human scalp and pig skin, with similar results for both species.

LAT encapsulation enhances its proliferative effects on keratinocytes, via MAPK signaling pathway. In conclusion, *ex vivo* and *in vitro* results indicate LAT-nanotransfersomes are a promising formulation for future *in vivo* clinical research as a therapy for hair growth disorders.

## Supporting Information Available

Short-term Western blots (PDF)
Videos S1 and S2: 3D video projection corresponding to Figure 3A,B (AVI, AVI)
Videos S3 and S4: 3D video projection corresponding to Figure 4A,B (AVI, AVI)

## Supplementary Material

mp2c00796_si_001.pdf

mp2c00796_si_002.avi

mp2c00796_si_003.avi

mp2c00796_si_004.avi

mp2c00796_si_005.avi
