# Supplementary material for: Latanoprost-Loaded Nanotransfersomes Designed for Scalp Administration Enhance Keratinocytes Proliferation
Source: Mol Pharm. 2022 Dec 12;20(5):2317–25. doi: 10.1021/acs.molpharmaceut.2c00796 (PMC10155202; doi:10.1021/acs.molpharmaceut.2c00796)
Supplement: Supplementary file 1 — mp2c00796_si_001.pdf [file mp2c00796_si_001.pdf]

Latanoprost-loaded nanotransfersomes designed for scalp administration enhance keratinocytes proliferation.

Eloy Pena-Rodríguez[a,β,‡,*], *Laura García-Vega*[b,c,d,‡], *Maria Lajarin Reinares*[a], *Marçal Pastor-Anglada* [b,c,d], *Sandra Pérez-Torras*[b,c,d#], *Francisco Fernandez-Campos*[a#,]

[a] Laboratory Reig Jofre, R&D Department, 08970 Sant Joan Despi, Barcelona, Spain

[b]Molecular Pharmacology and Experimental Therapeutics, Department of Biochemistry and Molecular. Biomedicine, Institute of Biomedicine (IBUB), University of Barcelona (IBUB), 08028 Barcelona, Spain

[c]Biomedical Research Networking Center in Hepatic and Digestive Diseases (CIBEREHD),

Carlos III Health Institute, 28029 Madrid, Spain

[d]Sant Joan de Déu Research Institute (IR SJD-CERCA) Esplugues de Llobregat, 08950 Barcelona, Spain

Suppplementary data:

Proliferation signalling was studied following 6 h and 24 h nanotransfersomes exposure. Non-stable signalling was observed at those time points.

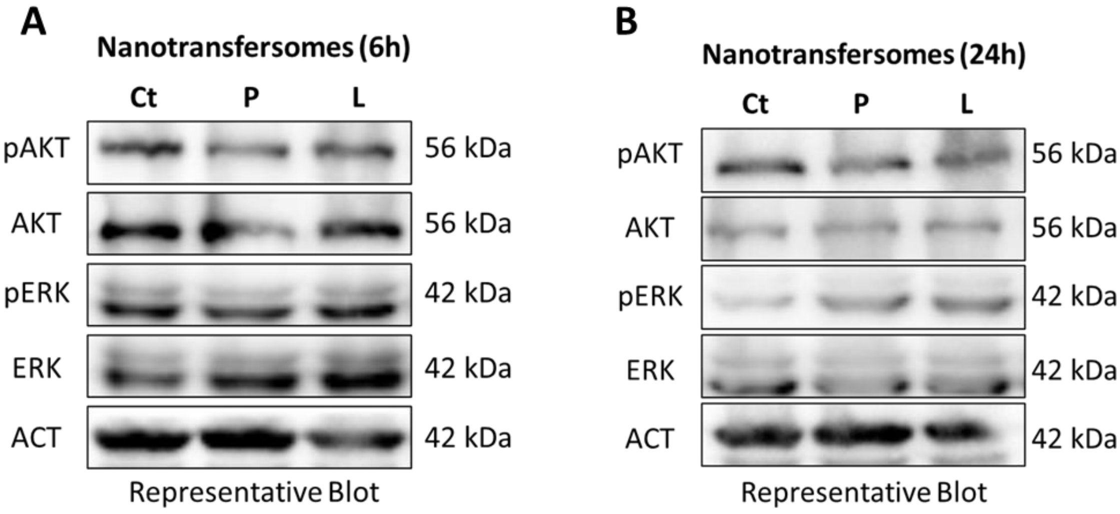

Figure S1. **Short LAT-nanotransfersomes treatment do not promote cell proliferation signalling**. A) 6 h treatment. B) 24 h treatments. Representative blots of nanotransfersomes effect on HaCaT cells. Ct: control, L: LAT-nanotransfersomes, P: Placebo- nanotransfersomes. Actin (ACT) was used as a loading control.
